# Taxonomic Novelties of Woody Litter Fungi (*Didymosphaeriaceae*, *Pleosporales*) from the Greater Mekong Subregion

**DOI:** 10.3390/biology11111660

**Published:** 2022-11-13

**Authors:** Guangcong Ren, Dhanushka N. Wanasinghe, Antonio Roberto Gomes de Farias, Kevin D. Hyde, Erandi Yasanthika, Jianchu Xu, Abhaya Balasuriya, Kandawatte Wedaralalage Thilini Chethana, Heng Gui

**Affiliations:** 1 School of Science, Mae Fah Luang University, Chiang Rai 57100, Thailand; 2 Center of Excellence in Fungal Research, Mae Fah Luang University, Chiang Rai 57100, Thailand; 3 School of Pharmacy, Guiyang Nursing Vocational College, Guiyang 550081, China; 4 Department of Economic Plants and Biotechnology, Yunnan Key Laboratory for Wild Plant Resources, Kunming Institute of Botany, Chinese Academy of Sciences, Kunming 650201, China; 5 Center for Mountain Futures, Kunming Institute of Botany, Chinese Academy of Sciences, Honghe 654400, China

**Keywords:** new taxa, *Ascomycota*, new genus, saprobic, taxonomy, phylogenetic

## Abstract

**Simple Summary:**

The Greater Mekong Subregion (GMS) has a diverse geographic landscape, and due to its varied environmental conditions, it harbors numerous florae, fauna, and microorganisms. Thus, the biodiversity in this region is exceptionally high. Over recent decades, the number of studies on microfungal diversity in the GMS increased rapidly. However, in the GMS the fungi of terrestrial habitats such as woody litter is still poorly researched. This paper introduces one monotypic genus, five novel species, and two new host records in Didymosphaeriaceae-inhabiting woody plant litter from the GMS and provides morpho-molecular justifications.

**Abstract:**

The Greater Mekong Subregion (GMS) is known as a diverse geographic landscape and one of the richest biodiversity hotspots in the world with a high fungal diversity. Collections were carried out in terrestrial habitats to determine the diversity of woody litter fungi in the GMS, with an emphasis on northern Thailand and the Yunnan Province of China. Morphological characteristics and multigene phylogenetic analyses of combined SSU, LSU, ITS, and *tef*1-α supported the placement of the new isolates in the family *Didymosphaeriaceae*. The phylogenetic affinities of our isolates are illustrated through maximum likelihood and Bayesian inference analyses. Seven species of woody litter fungi were identified, comprising a new monotypic genus, *Septofusispora*; five novel species (*Chromolaenicola sapindi*, *Dictyoarthrinium thailandicum*, *Karstenula lancangensis*, *Septofusispora thailandica*, and *Spegazzinia jinghaensis*); and new host records of two species (*Austropleospora archidendri*, and *Montagnula donacina*). Furthermore, this study provides a synopsis of the *Montagnula* aff. *donacina* species based on their morphological characteristics, which can be useful in the species-level identifications in this genus.

## 1. Introduction

The Greater Mekong Subregion (GMS) is a global biodiversity hotspot with a 2.5 million km^2^ land area [1], including Cambodia, Lao PDR, Myanmar, Thailand, the People’s Republic of China, and Vietnam. Due to its varied environmental conditions, the GMS harbors abundant biodiversity [2,3]. Numerous studies have shown that China (Yunnan Province) and Thailand have the potential to support a high diversity of macro- and micro-fungi, many yet to be discovered [3,4,5,6,7,8,9]. For instance, many saprobic taxa have been discovered on woody litter in this region [8,10,11,12,13,14,15,16,17,18,19,20,21]. Leaf litter and freshwater taxa have been well-studied in the GMS [5,7,22,23], but less attention has been given to saprobic fungi on woody litter in terrestrial habitats.

The *Didymosphaeriaceae* [24] is a diverse family of *Pleosporales*, comprising 33 genera [25]. Its species occur on a wide range of hosts in various habitats worldwide [26,27]. *Didymosphaeriaceae* species include endophytes, pathogens (plants and occasionally humans), and saprobes on woody branches, herbaceous stems, leaves, pods, and soil [28,29,30]. *Didymosphaeriaceae* comprises economically important fungi, such as the *Austropleospora* and *Barria* species, which have potential agricultural and medical applications, or the species of *Deniquelata,* which cause plant disease [29,31].

The sexual morphs of *Didymosphaeriaceae* are characterized by globose to sub-globose, central ostiolate ascomata; a peridium with several layers of lightly pigmented to dark brown or black cells of *textura angularis*; cellular or trabeculate pseudoparaphyses; 2–4-spored or 8-spored, bitunicate, fissitunicate, cylindric or oblong, pedicellate asci; and 1–2-seriate, overlapping, ellipsoid or oblong, 1–3-septate or muriform ascospores [29,30]. The asexual morphs are diverse, i.e., camarosporium-like, diplodia, fusicladium, pithomyces, phoma, and spegazzinia-like [32]. Out of the *Didymosphaeriaceae* genera, ten (*Alloconiothyrium*, *Cylindroaseptospora*, *Dictyoarthrinium*, *Neptunomyces*, *Paraconiothyrium*, *Paracamarosporium*, *Pseudocamarosporium*, *Pseudopithomyces*, *Spegazzinia,* and *Xenocamarosporium*) were introduced based on their asexual morphs characters only. *Alloconiothyrium* has pycnidial conidiomata with a single cavity and olivaceous-brown conidia [28]. *Cylindroaseptospora* has hyaline, cylindrical, aseptate conidia [33]. *Dictyoarthrinium* has square-to-spherical, subspherical or oblong, pale-to-dark brown, often four-celled conidia [34]. *Neptunomyces* has aseptate, golden yellow, subcylindrical conidia [29]. *Paraconiothyrium* has eustromatic conidiomata and hyaline-to-brown conidia [29]. *Paracamarosporium* has pale brown-to-brown, ellipsoid-to-ovoid, with obtuse ends, and 1–3 transversely septate conidia [35]. *Pseudocamarosporium* has oblong, muriform, brown-to-dark-brown conidia, with transverse, longitudinal, and oblique septa [36]. *Pseudopithomyces* has fusiform, verruculose dark conidia, producing brown-to-black colonies on the host [37]. *Spegazzinia* produces two types of conidia in the same mycelium: α conidia which are composed of 4–8 subglobose, dark cells with very long spines, while β conidia are subspherical or broadly ellipsoid conidia, in general flattened in one plane, crucially septate or muriform, pale brown and smooth [38]. Finally, *Xenocamarosporium* has ellipsoidal-to-subcylindrical, golden-brown, and verruculose conidia with (1–)3-septa [35].

On the other hand, twenty-three genera of *Didymosphaeriaceae* were introduced with their sexual morphs. *Austropleospora* has clavate-to-cylindrical, 6–8-spored asci and dictyosporous, ellipsoidal, and yellowish-brown ascospores [30,33]. *Barria* has short, knob-like pedicellate asci and brown, muriform ascospores [29]. *Bimuria* has fissitunicate, 2-spored asci with muriform, dark brown, and verrucose ascospores [29]. *Chromolaenicola* has cylindrical asci with an ocular chamber, and ellipsoid-to-broadly fusiform, muriform ascospores with three transverse septa and one vertical septum [39]. *Curreya* has small, sclerotial cells of its peridium, and narrower, thinner-walled asci [29]. *Deniquelata* has bitunicate asci and brown, muriform ascospores [31]. *Didymocrea* has unitunicate asci and two-celled, brown ascospores [31]. *Julella* has cylindric or oblong, 2-spored asci, and oblong-to-narrowly oblong, muriform ascospores [31]. *Didymosphaeria* has paraphyses richly anastomosing above the asci, and brown, thin, distoseptate ascospores [40]. *Kalmusia* has clavate asci, with narrowly ovoid to clavate, pale brown, and 3-septate ascospores [29]. *Kalmusibambusa* has multi-loculate ascostromata and cylindrical asci [41]. *Karstenula* has cylindrical-to-cylindro-clavate asci, with short, furcate pedicel, ellipsoid-to-fusoid, reddish-brown to dark brown, muriform ascospores [29]. *Laburnicola* has ellipsoidal-to-fusoid ascospores, with 6–8 transverse septa and 1–2 longitudinal septa [42]. *Letendraea* has obclavate-to-cylindrical asci and fusoid-to-oblong, 1-septate ascospores [31]. *Lineostroma* has trabeculate pseudoparaphyses asci with a short pedicel and 1-septate ascospores [31]. *Montagnula* has claviform asci, fusoid-or-ellipsoid ascospores with transverse septa and one or more longitudinal septa [31]. *Neokalmusia* has cylindric-clavate, 4–8-spored asci, and fusiform, yellowish-brown-to-reddish-brown, 3–5-septate ascospores with a sheath [31]. *Paramassariosphaeria* has cylindrical-clavate asci with a long pedicel, and curved-fusoid, asymmetrical ascospores with a mucilaginous sheath. [31] *Paraphaeosphaeria* has bitunicate asci with a short pedicel and multi-septate, broadly elliptical, yellowish-brown ascospores [42]. *Phaeodothis* has a sparse hamathecium, consisting of cellular pseudoparaphyses and 1-septate ascospores [31]. *Tremateia* has fissitunicate, clavate asci, and ellipsoid, muriform ascospores [29]. *Verrucoconiothyrium* has one-septate or aseptate, brown, subcylindrical-to-narrowly ellipsoid conidia [35]. Finally, *Vicosamyces* forms orange-brown wounds and 2-celled apiospores [29].

In our survey of the diversity of woody litter fungi in the GMS, the field collections were carried out within the Yunnan Province (China) and northern Thailand. This study aimed to (1) look for novel species and new host records supported by morphological illustrations and multi-gene phylogenetic analyses based on combined SSU, LSU, ITS, and *tef*1-α sequence data, and (2) provide a synopsis of the *Montagnula* species based on phylogeny and morphology.

## 2. Materials and Methods

### 2.1. Sample Collection, Morphological Observation, and Fungal Isolation

Decayed woody samples were collected from mixed forest areas located in Thailand (Chiang Mai, Chiang Rai, and Tak Provinces) during the wet season (August and September 2019), and in China (Yunnan Province) during the dry season (March 2020). The woody litter was cut into no more than 20 cm pieces. Collected samples were placed in separate zip-lock plastic bags and transported to the laboratory.

Specimens were examined using a stereomicroscope (Olympus SZ61, Tokyo, Japan). Micro-morphological characteristics were photographed using a Canon EOS 600D (Tokyo, Japan) digital camera mounted on a Nikon ECLIPSE 80i (Tokyo, Japan) compound microscope. All microscopic measurements were taken using the Tarosoft (R) Image Frame Work v.09 program, and the measurements were reported as minimum–maximum values and average values. Images were processed with Adobe Photoshop CS6 software v.13 (Adobe Systems, San Jose, CA, USA).

Single-spore isolation was used to obtain pure cultures. The ascomata containing ascospores were transferred using a sterile needle to a drop of sterile water on a flamed microscope slide. The spore suspension was spread over a few square centimeters of a Petri plate containing water agar (WA) or potato dextrose agar (PDA). Germinating spores were photographed, transferred to PDA media, and incubated at room temperature for seven days. Cultures were then photographed, and their characters recorded. After another week, hyphal tips were transferred into PDA plates and grown at 25 °C in the daylight [43]. Herbarium materials were deposited at the herbarium of Mae Fah Luang University, Chiang Rai Province, Thailand (MFLU), the Cryptogams Kunming Institute of Botany, Academia Sinica (HKAS), Kunming Institute of Botany, Chinese Academy of Sciences, China, and living cultures were deposited at the Culture Collection of Mae Fah Luang University (MFLUCC), Mae Fah Luang University, Thailand, and Kunming Institute of Botany Culture Collection (KUMCC), Kunming Institute of Botany, Chinese Academy of Sciences, China. Faces of fungi [44] and Index Fungorum [45] numbers were obtained for the new taxa, and the details were added to the Greater Mekong Subregion’s webpage [8].

### 2.2. DNA Extraction, PCR Amplification, and Sequencing

Fungal mycelia were scraped from the 14-day-old colonies grown on PDA at 25–30 °C, and the DNA was isolated using the Biospin Fungus Genomic DNA Extraction Kit (BioFlux^®^ Hangzhou, China). Polymerase chain reactions (PCRs) were conducted to amplify parts of the small nuclear ribosomal subunit rDNA (SSU), internal transcribed spacer region (ITS), large nuclear ribosomal subunit rDNA (LSU), and translation elongation factor 1-alpha gene (*tef*1-α) using primer pairs NS1/NS4 [46], ITS5/ITS4 [46], LR0R/LR5 [47], and EF1-983F/EF1-2218R [48], respectively. PCR was carried out in a 25 μL reaction volume containing 12.5 μL 2X PCR MasterMix (TIANGEN Co., Bejing, China), 8.5 μL double distilled water, 2 μL genomic DNA, and 1 μL of each primer. PCR thermal cycles for SSU, LSU, ITS, and *tef*1-α gene regions were conducted following Tennakoon et al. [49]. PCR products were sequenced at the Qingke Company, Yunnan Province, China.

### 2.3. Phylogenetic Analyses

Phylogenetic analyses were performed as described in Dissanayake et al. [50]. Each newly generated sequence was assembled using BioEdit 7.0.9.0 [51] and subjected to BLAST searches against the NCBI nucleotide non-redundant database (https://blast.ncbi.nlm.nih.gov/Blast.cgi (accessed on 20 August 2021)) for selection of the closest matching taxa. Based on BLAST search results and recently published data, sequences of representative taxa were downloaded and used for comparison [30,33,34,52] (Table 1). Individual gene regions were aligned using MAFFT v.7 (http://mafft.cbrc.jp/alignment/server/ (accessed on 20 May 2022)) [53], and the uninformative gaps and ambiguous regions were manually removed and different gene regions were concatenated using BioEdit 7.0.9.0.

The ML analysis was performed on the CIPRES Science Gateway v.3.3 (http://www.phylo.org/portal2/ (accessed on 21 May 2022), [54]) using RAxML-HPC2 on XSEDE v.8.2.12 [55] with parameters adjusted for 1000 bootstrap iterations and the GTRGAMMA substitution model. Gaps were treated as missing data, and the branches of zero length were collapsed [56]. Bayesian inference was performed in MrBayes v.3.2.2 using Markov chain Monte-Carlo sampling (BMCMC) [57] to determine posterior probabilities (PPs) [58,59]. The model of evolution was estimated using MrModeltest v.2.3 [60] via PAUP v.4.0b10 [61]. Six simultaneous Markov chains were run for 2,000,000 generations, with trees sampled every 200 generations, until it was stopped when the standard deviation of split frequencies between the two simultaneous runs dropped below 0.01. The first 25% of sampled trees was discarded as part of the burn-in procedure, and the remaining 7501 trees were used to calculate posterior probabilities in the consensus tree. Phylogenetic trees were visualized with FigTree v.1.4.0 [62] and edited using Microsoft PowerPoint and Adobe Illustrator^®^ CS6 v.26.0 (Adobe Systems, San Jose, CA, USA). The newly produced sequences were deposited in the GenBank nucleotide database (Table 1).

**Table 1:** Taxa used in the phylogenetic analysis, species voucher/culture numbers, and GenBank accession numbers for the sequences.

Taxon	Strain Number	GenBank Accession Numbers				Reference
		SSU	LSU	ITS	*tef*1-α	
*Alloconiothyrium aptrootii*	CBS 980.95 ^T^	JX496121	JX496234	NA	NA	[28]
*A. aptrootii*	CBS 981.95 ^T^	JX496122	JX496235	NA	NA	[28]
*A. camelliae*	NTUCC 17-032-1 ^T^	MT112294	MT071270	MT071221	MT232967	[63]
*A. camelliae*	NTUCC 17-032-2	MT112295	MT071271	MT071222	MT232965	[63]
*A. camelliae*	NTUCC 17-032-3	MT112296	MT071272	MT071223	MT232966	[63]
*A. encephalarti*	CPC: 35980	MN562102	MN567610	NA	NA	[63]
*Austropleospora archidendri*	MFLUCC 17-2429	MK347757	MK347974	MK347863	MK360044	[33]
*A. archidendri*	CBS 168.77	NA	JX496162	JX496049	NA	[28]
***A. archidendri***	**KUMCC 21-0680**	**OP059006**	**OP059055**	**OP058964**	**OP135941**	**This study**
*A. keteleeriae*	MFLUCC 18-1551 ^T^	MK347802	MK348021	MK347910	MK360045	[33]
*A. ochracea*	KUMCC 20-0020 ^T^	MT799859	MT799860	MT808321	MT872714	[30]
*A. osteospermi*	BRIP51628	FJ481946	NA	NA	NA	[64]
*Bambusistroma didymosporum*	MFLU 15-0057 ^T^	KP761733	KP761730	KP761737	KP761727	[65]
*B. didymosporum*	MFLU 15-0058	KP761734	KP761731	KP761738	KP761728	[65]
*Bimuria novae-zelandiae*	CBS 107.79 ^T^	MH861181	AY016356	AY016338	DQ471087	[66]
*Chromolaenicola chiangraiensis*	MFLUCC 17-1493 ^T^	MN325017	MN325005	MN325011	MN335650	[39]
*C. clematidis*	MFLUCC 17-2075 ^T^	MT310601	MT214554	MT226671	NA	[67]
*C. lampangensis*	MFLUCC 17-1462 ^T^	MN325016	MN325004	MN325010	MN335649	[39]
*C. thailandensis*	MFLUCC 17-1510 ^T^	MN325018	MN325006	MN325012	MN335651	[39]
*C. thailandensis*	MFLUCC 17-1475	MN325019	MN325007	MN325013	MN335652	[39]
*C. nanensis*	MFLUCC 17-1473 ^T^	MN325015	MN325003	MN325009	MN335648	[39]
*C. nanensis*	MFLUCC 17-1477	MN325014	MN325002	MN325008	MN335647	[39]
*C. siamensis*	MFLUCC 17-2527 ^T^	NR_163337	NG_066311	NA	NA	[33]
***C. sapindi***	**KUMCC 21-0564 ^T^**	**OP059009**	**OP059058**	**OP058967**	**OP135943**	**This study**
***C. sapindi***	**KUMCC 21-0594**	**OP059010**	**OP059059**	**OP058968**	**OP135944**	**This study**
*Cylindroaseptospora leucaenae*	MFLUCC 17-2424 ^T^	NR_163333	NG_066310	MK347856	MK360047	[33]
*C. siamensis*	MFLUCC 17-2527 ^T^	MK347760	MK347976	MK347866	MK360048	[33]
*Deniquelata barringtoniae*	MFLUCC 11-0422 ^T^	NR_111779	NG_042696	JX254656	NA	[68]
*D. vittalii*	NFCCI4249 ^T^	MF406218	MF182395	MF622059	MF182398	[69]
*Dictyoarthrinium hydei*	SQUCC 13296 ^T^	MW077145	NA	MW077161	MW075771	[26]
*D. musae*	MFLUCC 20-0105 ^T^	MT482323	MT482320	MT482326	MT495602	[52]
*D. musae*	MFLUCC 20-0106 ^T^	MT482324	MT482321	MT482327	MT495603	[52]
*D. sacchari*	MFLUCC 20-0107	MT482325	MT482322	MT482328	NA	[52]
*D. sacchari*	CBS 529.73	NA	MH872479	NA	NA	[70]
***D. thailandicum***	**KUMCC 21-0664 ^T^**	**OP059007**	**OP059056**	**OP058965**	NA	**This study**
***D. thailandicum***	**KUMCC 21-0665**	**OP059008**	**OP059057**	**OP058966**	**OP135942**	**This study**
*Didymocrea sadasivanii*	CBS 438.65 ^T^	MH858658	DQ384103	NA	NA	[70]
*Didymosphaeria rubi-ulmifolii*	MFLUCC 14-0023 ^T^	NA	KJ436586	NG_063557	NA	[40]
*D. rubi-ulmifolii*	MFLUCC 14-0024	NA	KJ436585	KJ436587	NA	[40]
*Kalmusia italica*	MFLUCC 14-0560 ^T^	KP325440	KP325441	KP325442	NA	[71]
*K. variispora*	CBS 121517 ^T^	MH863113	MH874668	NA	NA	[28]
*K. ebuli*	CBS 123120 ^T^	KF796674	JN644073	JN851818	NA	[72]
*Kalmusibambusa triseptata*	MFLUCC 13-0232 ^T^	KY682697	KY682695	KY682696	NA	[41]
***Karstenula lancangensis***	**KUMCC 21-0670 ^T^**	**OP059011**	**OP059060**	**OP058969**	NA	**This study**
***K. lancangensis***	**KUMCC 21-0677**	**OP059012**	**OP059061**	**OP058970**	NA	**This study**
*K. rhodostoma*	CBS 690.94	NA	GU301821	GU296154	GU349067	[73]
*K. rhodostoma*	CBS 691.94	LC014559	AB807531	AB797241	AB808506	[73]
*Laburnicola hawksworthii*	MFLUCC 13-0602 ^T^	KU743194	KU743195	KU743196	NA	[42]
*L. muriformis*	MFLUCC 14-0921 ^T^	KU743200	KU743201	KU743202	NA	[42]
*Letendraea cordylinicola*	MFLUCC 11-0150	KM213996	KM213999	KM214002	NA	[40]
*L. cordylinicola*	MFLUCC 11-0148 ^T^	NR_154118	NG_059530	KM214001	NA	[40]
*Montagnula aloes*	CPC 19671 ^T^	JX069863	JX069847	NA	NA	[74]
*M. aloes*	CBS 132531 ^T^	NR_111757	NG_042676	NA	NA	[74]
*M. appendiculata*	CBS 109027 ^T^	DQ435529	AY772016	NA	NA	[75]
*M. bellevaliae*	MFLUCC 14-0924 ^T^	KT443906	KT443902	KT443904	NA	[76]
*M. camporesii*	MFLUCC 16-1369 ^T^	MN401746	NG_070946	NG_068418	MN397908	[77]
*M. chiangraiensis*	MFLUCC 17-1420 ^T^	NR_168864	NG_068707	NG_070155	NA	[39]
*M. chromolaenae*	MFLUCC 17-1435 ^T^	NR_168865	NG_068708	NG_070156	NA	[39]
*M. chromolaenicola*	MFLUCC 17-1469 ^T^	NR_168866	NG_070948	NG_070157	MT235773	[39]
*M. cirsii*	MFLUCC 13-0680	KX274242	KX274249	KX274255	KX284707	[78]
*M. cylindrospora*	UTHSC: DI16-208 ^T^	LT796834	LN907351	NA	LT797074	[74]
*M. donacina*	HFG07004	MF967419	MF183940	NA	NA	[79]
*M. donacina*	HVVV01	KJ628375	KJ628377	KJ628376	NA	[80]
***M. donacina***	**KUMCC 21-0653**	**OP059003**	**OP059052**	**OP058961**	**OP135938**	**This study**
***M. donacina***	**KUMCC 21-0579**	**OP059005**	**OP059054**	**OP058963**	**OP135940**	**This study**
***M. donacina***	**KUMCC 21-0631**	**OP059004**	**OP059053**	**OP058962**	**OP135939**	**This study**
*M. graminicola*	MFLUCC 13-0352 ^T^	KM658314	KM658315	KM658316	NA	[81]
*M. jonesii*	MFLUCC 16-1448 ^T^	KY313619	KY273276	KY313618	KY313620	[49]
*M. krabiensis*	MFLUCC 16-0250 ^T^	NR168179	NG068826	NG068385	MH412776	[82]
*M. puerensis*	KUMCC 20-0225 ^T^	MW567739	MW575866	MW575864	MW575859	[83]
*M. puerensis*	KUMCC 20-0331	MW567740	MW575867	MW575865	MW575860	[83]
*M. saikhuensis*	MFLUCC 16-0315 ^T^	KU743209	KU743210	KU743211	NA	[42]
*M. scabiosae*	MFLUCC 14-0954 ^T^	KT443907	KT443903	KT443905	NA	[76]
*M. thailandica*	MFLUCC 17-1508 ^T^	MT214352	NG070949	NG070158	MT235774	[39]
*Neokalmusia brevispora*	KT 1466 ^T^	LC014573	AB524600	AB524459	AB539112	[73]
*N. scabrispora*	KT 1023	LC014575	AB524593	AB524452	AB539106	[73]
*Neptunomyces aureus*	CMG12 ^T^	MK912121	NA	NA	MK948000	[84]
*N. aureus*	CMG13	MK912122	NA	NA	MK948001	[84]
*Paraconiothyrium cyclothyrioides*	CBS 972.95 ^T^	JX496119	JX496232	AY642524	NA	[28]
*P. cyclothyrioides*	CBS 432.75	MH860933	MH872689	NA	NA	[28]
*P. estuarinum*	CBS 109850	MH862842	MH874432	NA	NA	[28]
*Paracamarosporium fagi*	CPC 24890	KR611886	KR611904	NA	NA	[35]
*P. fagi*	CPC 24892 ^T^	KR611887	KR611905	NA	NA	[35]
*Paramassariosphaeria anthostomoides*	CBS 615.86	MH862005	GU205223	GU205246	NA	[28]
*P. anthostomoides*	MFLU 16-0172 ^T^	KU743206	KU743207	KU743208	NA	[42]
*Paraphaeosphaeria rosae*	MFLUCC 17-2547	MG828935	MG829044	MG829150	MG829222	[85]
*P. rosae*	MFLUCC 17-2549 ^T^	MG828937	MG829046	MG829152	MG829223	[85]
*P. rosicola*	MFLUCC 15-0042 ^T^	NR_157528	MG829047	MG829153	NA	[85]
*Phaeodothis winteri*	CBS 182.58	NA	GU301857	GU296183	NA	[86]
*Pseudocamarosporium propinquum*	MFLUCC 13-0544	KJ747049	KJ813280	KJ819949	NA	[36]
*P. pteleae*	MFLUCC 17-0724 ^T^	NR_157536	MG829061	MG829166	MG829233	[85]
*Pseudopithomyces entadae*	MFLUCC 17-0917 ^T^	NA	NG_066305	MK347835	MK360083	[33]
*P. rosae*	MFLUCC 15-0035 ^T^	MG828953	MG829064	MG829168	NA	[85]
***Septofusispora thailandica***	**KUMCC 21-0647 ^T^**	**OP059013**	**OP059062**	**OP058971**	**OP135945**	**This study**
***S. thailandica***	**KUMCC 21-0652**	**OP059014**	**OP059063**	**OP058972**	NA	**This study**
*Spegazzinia bromeliacearum*	URM 8084 ^T^	MK804501	MK809513	NA	NA	[87]
*S. deightonii*	MFLUCC 20-0002 ^T^	MN956768	MN956772	MN956770	MN927133	[73]
*S. intermedia*	CBS 249.89 ^T^	MH862171	MH873861	NA	NA	[70]
***S. jinghaensis***	**KUMCC 21-0495 ^T^**	**OP059015**	**OP059064**	**OP058973**	**OP135946**	**This study**
***S. jinghaensis***	**KUMCC 21-0496**	**OP059016**	**OP059065**	**OP058974**	**OP135947**	**This study**
*S. lobulata*	CBS 361.58 ^T^	MH857812	MH869344	NA	NA	[70]
*S. musae*	MFLUCC 20-0001 ^T^	MN930512	MN930514	MN930513	MN927132	[52]
*S. neosundara*	MFLUCC 15-0456 ^T^	KX965728	KX954397	KX986341	NA	[41]
*S. radermacherae*	MFLUCC 17-2285 ^T^	MK347740	MK347957	MK347848	MK360088	[33]
*S. tessarthra*	SH 287	JQ673429	AB807584	AB797294	AB808560	[73]
*Tremateia arundicola*	MFLU 16-1275 ^T^	KX274241	KX274248	KX274254	KX284706	[49]
*T. guiyangensis*	GZAAS01 ^T^	KX274240	KX274247	KX274253	KX284705	[49]
*T. murispora*	GZCC 18-2787 ^T^	NR_165916	MK972751	MK972750	MK986482	[88]
*Verrucoconiothyrium nitidae*	CBS: 119209	EU552112	EU552112	NA	NA	[89]
*Xenocamarosporium acaciae*	CBS: 139895 ^T^	NR_137982	NG_058163	NA	NA	[35]
*X. acaciae*	MFLUCC 17-2432	MK347766	MK347983	MK347873	MK360093	[33]

The newly generated sequences are indicated in bold. ^T^ refers to ex-type strains and NA refers to “no data in GenBank”.

## 3. Results

### 3.1. Phylogeny

The combined dataset of SSU, LSU, ITS, and *tef*1-α comprised 109 strains of *Didymosphaeriaceae* and 2 strains of *Periconia didymospora* (MFLU 15-0057 and MFLU 15-0058), the latter 2 strains from *Periconiaceae* as the outgroup taxa (Table 1). The final concatenated aligned data matrix had 2939 characters (SSU: 909 bp, LSU: 725 bp, ITS: 382 bp, and *tef*1-α: 923 bp), including alignment gaps. The RAxML analysis of the combined dataset yielded the best-scoring tree with a final ML optimization likelihood value of −18,003.440225. The matrix had 891 distinct alignment patterns with 24.94% undetermined characters or gaps. The estimated base frequencies were as follows: A = 0.235809, C = 0.252488, G = 0.274923, T = 0.236780; substitution rates: AC = 1.104424, AG = 2.404211, AT = 1.383490, CG = 1.027352, CT = 6.936114, GT = 1.00; and gamma distribution shape parameter: α = 0.187954 and tree-length = 2.009896.

**Figure 1:**
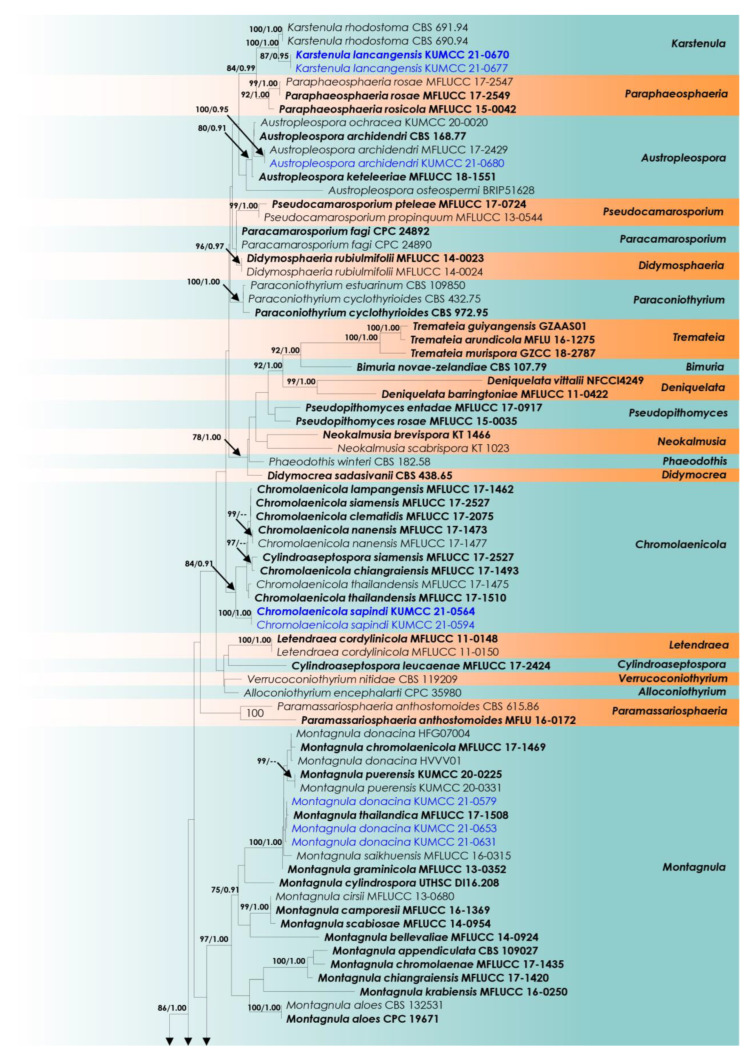

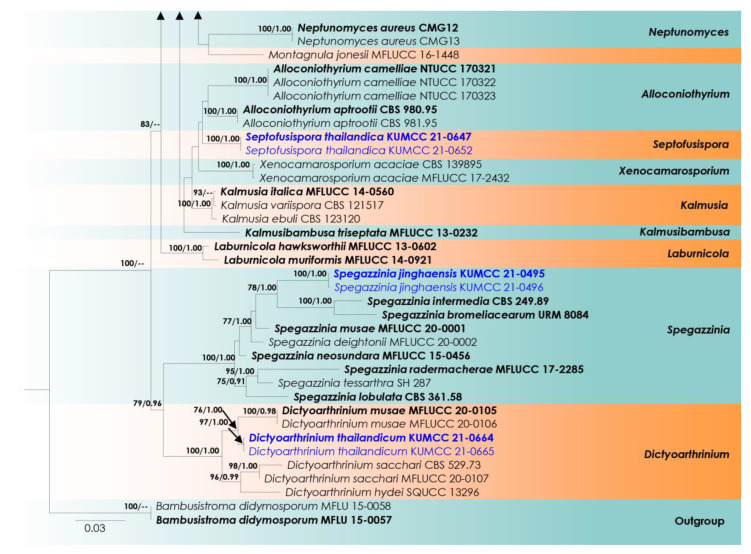
Phylogram generated from ML analysis based on the combined SSU, LSU, ITS, and *tef*1-α dataset. Bootstrap support values for ML equal to or higher than 75%, and BYPP equal to or greater than 0.90 are shown above the nodes. The ex-type strains are in bold, and new isolates are in blue. The tree is rooted with *Periconia didymospora* (MFLU 15-0057 and MFLU 15-0058).

*Didymosphaeriaceae* comprises 33 genera, but molecular data are available only for 28 of them. Thus, sequence data representing 28 genera were used in the phylogenetic analyses. Phylogenetic trees resulting from ML and BI (Figure 1) analyses have similar overall topologies compared to the trees illustrated in Dissanayake et al. [30], Jayasiri et al. [33], and Samarakoon et al. [34]. These results show that the KUMCC 21-0647 and KUMCC 21-0652 isolates formed a monophyletic clade independent from all others (*Alloconiothyrium*, *Kalmusia*, and *Xenocamarosporium*) (Figure 1), and is thus introduced as a new genus, *Septofusispora*, with *Septofusispora thailandica* as the type species. *Karstenula lancangensis* (KUMCC 21-0670, KUMCC 21-0677) was clustered sister to the type species of this genus, *K. rhodostoma* (CBS 690.94, CBS 691.94), with 100% ML bootstrap and 1.00 BYPP statistical support (Figure 1). *Chromolaenicola sapindi* (KUMCC 21-0564, KUMCC 21-0594) was grouped in an independent lineage inside *Chromolaenicola* with 84% ML bootstrap and 0.91 BYPP support (Figure 1). *Spegazzinia jinghaensis* (KUMCC 21-0495, KUMCC 21-0496) has a sister affiliation to *S. bromeliacearum* (URM 8084) and *S. intermedia* (CBS 249.89) with 78% ML bootstrap and 1.00 BYPP support (Figure 1). *Dictyoarthrinium thailandicum* (KUMCC 21-0664, KUMCC 21-0665) was nested with *D. musae* (MFLUCC 20-0105 and MFLUCC 20-0106) with 76% ML bootstrap and 1.00 BYPP support (Figure 1). The samples of *Montagnula donacina* (KUMCC 21-0579, KUMCC 21-0653, and KUMCC 21-0631) were grouped with eight *Montagnula* species, *viz.*, *M. chromolaenicola* (MFLUCC 17-1469), *M. donacina* (HFG07004 and HVVV01), *M. puerensis* (KUMCC 20-0225 and KUMCC 20-0331), *M. saikhuensis* (MFLUCC 16-0315), *M. thailandica* (MFLUCC 17-1508), and *M. graminicola* (MFLUCC 13-0352) in a monophyletic clade (Figure 1), while *A. archidendri* (KUMCC 21-0680) formed a well-supported clade with other strains of this genus with 80% ML bootstrap and 0.91 BYPP support. Based on the nucleotide base pair comparisons of LSU and ITS, our new strains are identical to the type strain of *Austropleospora archidendri* (CBS 168.77) with 100% similarity (Figure 1), but they appear in a different branch, perhaps due to the lack of homologous SSU and *tef*1-α sequences from the type collection.

### 3.2. Taxonomy

***Septofusispora*** G.C. Ren and K.D. Hyde, gen. nov.

Index Fungorum number: IF559804; FacesofFungi number: FoF 10702.

*Etymology*: Epithet refers to the fusiform and septate spores of this genus.

*Saprobic* on decaying wood. Sexual morph: *Ascomata* solitary or gregarious, erumpent to immersed, globose-to-subglobose, black. *Ostiole* central. *Peridium* thick, comprising pale-to-brown cells of *textura angularis*. *Hamathecium* of septate, branched, cellular pseudoparaphyses. *Asci* 8-spored, bitunicate, fissitunicate, cylindrical-to-clavate, with or without an ocular chamber, with a pedicel. *Ascospores* overlapping, brown, fusiform, with 4–5 transverse septa, smooth-walled, guttulate. Asexual morph: undetermined.

Notes: In our phylogenetic analysis, *Septofusispora* is seemingly nested near *Alloconiothyrium*, *Kalmusibambusa*, *Kalmusia*, and *Xenocamarosporium* (Figure 1), but it is well-separated from these genera. *Septofusispora* has uni-loculate ascomata, clavate asci, fusiform, guttulate ascospores with 4–5 transverse septa, whereas the *Kalmusia* species have ovoid-to-clavate asci, ovoid-to-clavate, 3-septate ascospores (sometimes muriform), with a mucilaginous sheath [81,90,91]. *Kalmusibambusa* has multi-loculate, elongate ascostromata, cylindrical asci, ellipsoidal-to-fusiform, 3-septate ascospores with round-to-acute ends and a wide mucilaginous sheath [41]. *Alloconiothyrium* and *Xenocamarosporium* are known only from their asexual morphs [28,63]. Most studies used molecular data to delimit species boundaries, which is not possible using morphological characters. Therefore, considering the morphological differences and phylogenetic support, we introduce *Septofusispora* as a new genus.

Type species: *Septofusispora thailandica* G.C. Ren and K.D. Hyde.

**Figure 2:**
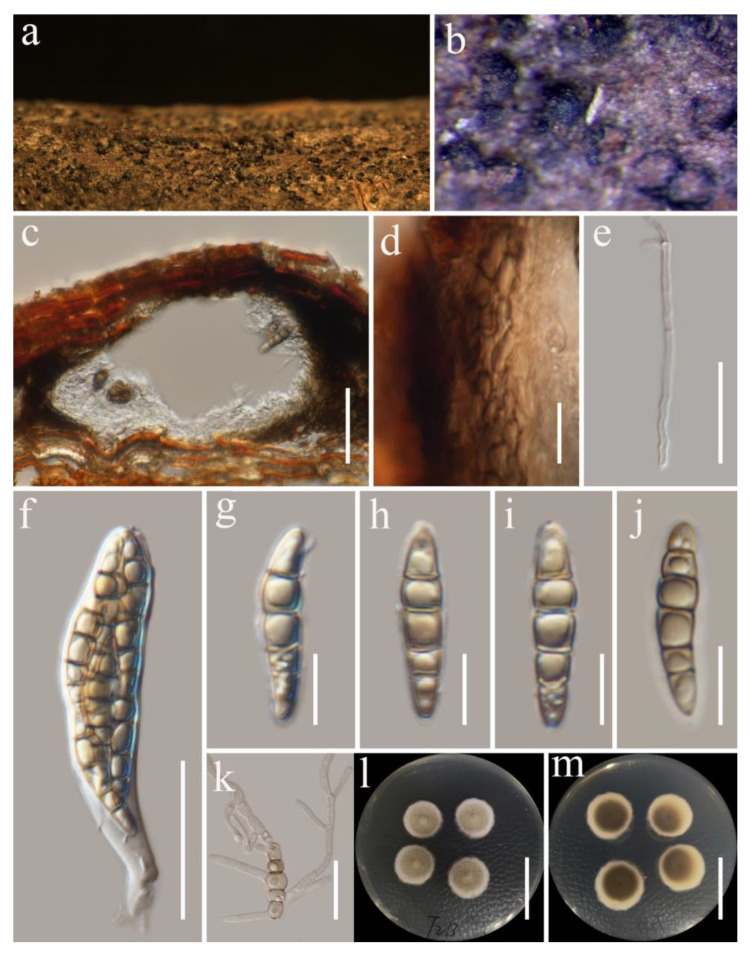
*Septofusispora thailandica* (MFLU 22-0043, holotype). (**a**,**b**) Appearance of ascomata on host substrate; (**c**) section of ascoma; (**d**) peridium; (**e**) hamathecium; (**f**) asci; (**g**–**j**) ascospores; (**k**) germinated ascospore; (**l**,**m**) culture characters on PDA (**l** from above, **m** from below). Scale bars, (**c**) 50 μm; (**d**,**e**,**k**) 20 μm; (**f**) 30 μm; (**g**–**j**) 10 μm; (**l**,**m**) 30 mm.

***Septofusispora thailandica*** G.C. Ren and K.D. Hyde, sp. nov. Figure 2.

Index Fungorum number: IF559805. FacesofFungi number: FoF 10703.

*Etymology*: The epithet reflects Thailand, where this species was collected.

Holotype: MFLU 22-0043.

*Saprobic* on dead woody twigs of *Castanopsis* sp. Sexual morph: *Ascomata* 140–190 × 155–210 μm ($\bar{x}$ = 165 × 180 μm, *n* = 5), solitary, scattered, erumpent-to-immersed, uni-loculate, globose-to-sub-globose, black. *Ostiole* central. *Peridium* 20–35 μm wide, thick, comprising 3–5 layers of light-brown-to-brown cells of *textura angularis*. *Hamathecium* of sparse, 1–2 μm wide, cylindrical, septate, branched, cellular pseudoparaphyses. *Asci* 60–80 × 12–16 μm ($\bar{x}$ = 73.9 × 14.9 μm, *n* = 20), 8-spored, bitunicate, fissitunicate, clavate, slightly broad at center, apically rounded, with short, rounded pedicel. *Ascospores* 24–26.5 × 5–6 μm ($\bar{x}$ = 25.5 × 5.3 μm, *n* = 30), overlapping uni- to bi-seriate, pale brown, narrowly fusiform, cell above median septum slightly wider than below, tapering towards ends, slightly acute at both ends, with 4–5 transverse septa, constricted at the septa, smooth-walled, guttulate, without a mucilaginous sheath. Asexual morph: Undetermined.

Culture characteristics: The colonies on PDA, reaching 15–20 mm diam. at 14 days at room temperature (25–30 °C), superficial, entire margin, umbonate at center, rough surface, with dense mycelia, velvety, raised, gray at the center, white at the edge; reverse atrovirens, darkening towards center and white at the edge.

Material examined: Thailand, Tak Province, Mogro Amphoe Umphang, on dead woody twigs of *Castanopsis* sp., 20 August 2019, G.C. Ren, T213 (MFLU 22-0043 holotype), ex-type culture KUMCC 21-0647; *ibid.*, T214 (MFLU 22-0044, isotype), living culture KUMCC 21-0652.

***Austropleospora*** R.G. Shivas and L. Morin, Fungal Diversity 40: 70 (2010).

*Austropleospora* was introduced by Morin et al. [64], with *A. osteospermi* as the type species. Ariyawansa et al. [37] transferred *Austropleospora* from *Pleosporaceae* to *Didymosphaeriaceae*, and four taxa are currently accepted [45]. *Austropleospora osteospermi* was introduced with both sexual and asexual morphs, but *A. archidendri* and *A. keteleeriae* were introduced with only their asexual morphs, while *A. ochracea* was introduced with only its sexual morph [30,33,64,92]. The *Austropleospora* species have been reported from Australia, China, Myanmar, and Thailand [28,30,33,64,92]. The species in the genus are saprobic on *Archidendron bigeminum*, *Leucaena* sp., *Keteleeria forturei*, and pathogenic on stems of *Chrysanthemoides monilifera* [28,33].

***Austropleospora archidendri*** (Verkley, Göker, and Stielow) Ariyaw. and K.D. Hyde, Fungal Diversity 75: 64 (2015) Figure 3.

≡ *Paraconiothyrium archidendri* Verkley, Göker and Stielow, Persoonia 32: 37 (2014).

Index Fungorum number: IF551419; FacesofFungi number: FoF 00936.

*Saprobic* on dead woody twigs of *Euphoria longana*. Sexual morph: Undetermined. Asexual morph: *Coelomycetous*. *Conidiomata* 165–225 µm high × 115–165 µm diam. ($\bar{x}$ = 190 × 140 µm, *n* = 10), scattered, immersed, unilocular, coriaceous, globose-to-sub-globose, brown-to-dark-brown with a central ostiole. *Ostiole* 60–75 × 40–50 μm ($\bar{x}$ = 70 × 45 μm, *n* = 5), short papillate, black. *Conidiomatal wall* 15–25 µm thick, 3–4-layered, composed of brown outer and hyaline inner layers, thin-walled cells of *textura angularis*. *Conidiophores* reduced into conidiogenous cells. *Conidiogenous cells* 3.7–6 × 2.8–3.8 μm ($\bar{x}$ = 4.2 × 3.4 μm, *n* = 10), enteroblastic, phialidic, determinate, discrete, doliiform-to-ampulliform, hyaline, smooth-walled, arising from stratum. *Conidia* 4.8–5.8 × 3–3.6 μm ($\bar{x}$ = 5.4 × 3.4 μm, *n* = 30), straight, initially hyaline, guttulate, becoming brown at maturity, sub-globose to ovate, one-celled, rounded ends, thick-walled.

Culture characteristics: The colonies reached 70–80 mm diam. on PDA at 14 days at room temperature (25–30 °C), superficial, flat, circular, medium-dense, rough, fluffy, zonate, raised between margin and center, gray at the margin, white at the center; reverse, zonate, pale gray at the margin, dark gray at the center and zonate.

Material examined: Thailand, Chiang Mai Province, Yang Piang Omkoi, on dead woody twigs of *Euphoria longana*, 25 August 2019, G.C. Ren, YP03 (MFLU 22-0042), living culture KUMCC 21-0680.

Known distribution: on leaf spot in *Archidendron bigeminum* (Myanmar), decaying pod of *Leucaena* sp. (Thailand) [28,33].

Notes: *Austropleospora archidendri* was introduced by Ariyawansa et al. [92] as a new combination of *Paraconiothyrium archidendri* based on the combined phylogeny of LSU, SSU, *β*-tubulin, and ITS sequence data. In the present study, the multi-gene phylogenetic analyses indicated that our new strain, KUMCC 21-0680, formed a sister clade with *A. archidendri* (MFLUCC 17-2429) with 100% ML bootstrap and 0.95 BYPP support (Figure 1). Since the type species lacks SSU and *tef*1-α sequences, the nucleotide base pair comparisons of LSU and ITS demonstrated that our new strains are identical to the type of the *Austropleospora archidendri* strain and other species (Table 1). Our strain, KUMCC 21-0680, is similar to *A. archidendri* (CBS 168.77, MFLUCC 17-2429) in having doliiform conidiogenous cells and sub-globose-to-ovate, brown, aseptate conidia [28,33]. *Austropleospora archidendri* was reported as a pathogen on *A. bigeminum* leaves in Thailand and a saprobe on the pods of a *Leucaena* sp. in Myanmar [28,33]. Therefore, we report our strain KUMCC 21-0680 as a new record of *A. archidendri* on woody litter of *Euphoria longana* in Thailand.

***Chromolaenicola*** Mapook and K.D. Hyde, Fungal Diversity 101: 20 (2020).

Chromolaenicola was introduced in Didymospheriaceae by Mapook et al. [39], with C. nanensis as the type species. Currently, Chromolaenicola comprises six species [67]: *Chromolaenicola chiangraiensis*, *C. clematidis*, *C. lampangensis*, and *C. siamensis*, which were reported from their asexual morphs, while *C. nanensis* and *C. thailandensis* were reported from their sexual morphs. The taxa of *Chromolaenicola* have only been reported so far from Thailand as saprobes on dead stems of *Chromolaena odorata*, *Clematis subumbellata*, and *Leucaena sp*. [33,39,67]. Here, we introduce a new sexual morph, *C. sapindi*, from China based on phylogenetic analyses and morphological evidence.

**Figure 4:**
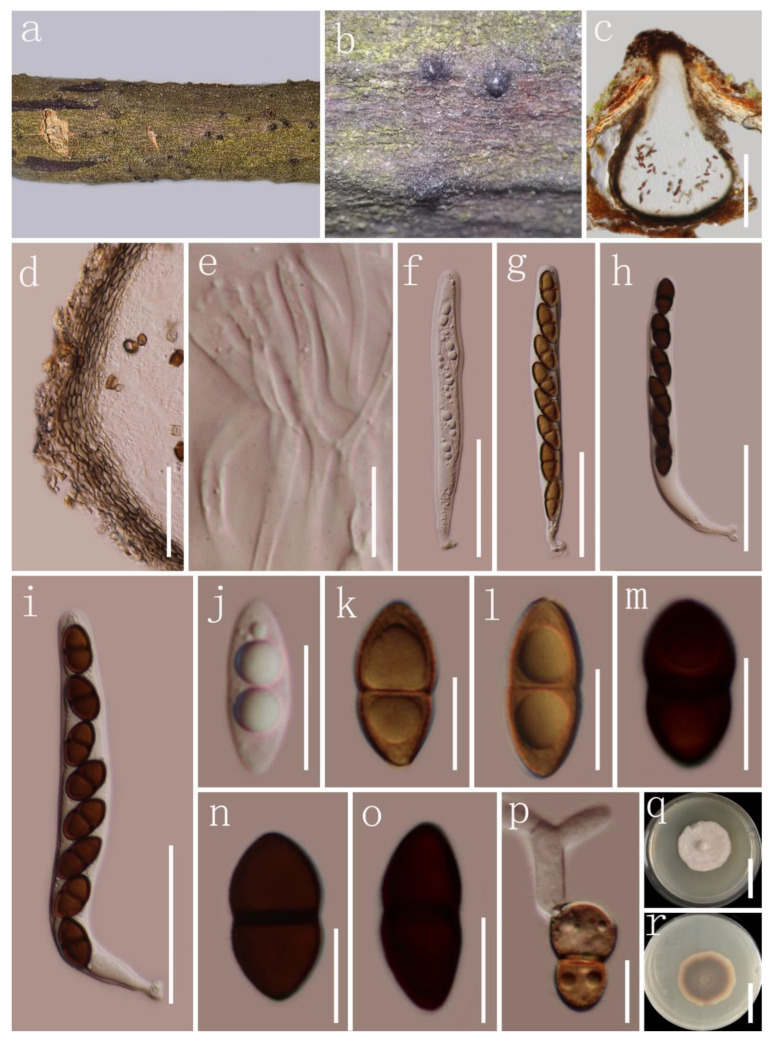
*Chromolaenicola sapindi* (HKAS 122789, holotype). (**a**,**b**) Appearance of ascomata on host substrate; (**c**) section of ascoma; (**d**) peridium; (**e**) hamathecium; (**f**–**i**) asci; (**j**–**o**) ascospores; (**p**) germinated ascospore; (**q**,**r**) culture characters on PDA (**q** from above, **r** from below). Scale bars, (**c**) 200 μm; (**d**,**f**–**i**) 50 μm; (**e**,**j**–**p**) 10 μm; (**q**,**r**) 30 mm.

***Chromolaenicola sapindi*** G.C. Ren and K.D. Hyde, sp. nov. Figure 4.

Index Fungorum number: IF559806. FacesofFungi number: FoF 10704.

*Etymology*: The epithet refers to the host genus *Sapindus*.

Holotype: HKAS 122789.

*Saprobic* on dead woody twigs of *Sapindus rarak*. Sexual morph: *Ascomata* 420–530 µm high × 270–350 µm diam. ($\bar{x}$ = 480 × 300 µm, *n* = 5), immersed-to-erumpent, solitary or scattered, coriaceous, ampulliform or obovoid, dark brown. *Ostiole* central. *Peridium* 15–25 µm thick, 4–7-layered, comprising pale-brown-to-brown cells of *textura angularis*. *Hamathecium* 1.5–3 µm wide, comprising cylindrical, septate, branching pseudoparaphyses, embedded in a hyaline, gelatinous matrix. *Asci* 125–155 × 12–16 µm ($\bar{x}$ = 138 × 13 µm, *n* = 20), bitunicate, 8-spored, cylindrical-clavate, straight, slightly curved at the end, apically rounded, with a pedicel (7–10 µm long). *Ascospores* 16–23 × 6.5–9.5 µm ($\bar{x}$ = 18.9 × 8 µm, *n* = 30), overlapping 1-seriate, ellipsoidal, initially hyaline-to-pale-brown and aseptate or 1-septate, guttulate, becoming reddish-brown-to-brown, and 1-septate at maturity, slightly constricted at the central septum, with or without guttules, thick and smooth-walled, without a gelatinous sheath. Asexual morph: undetermined.

Culture characteristics: The colonies on PDA reached 20–30 mm diam. after 14 days at room temperature (25–30 °C), superficial, circular, umbonate at the center, with dense mycelia, smooth, downy, velvety, fimbriate, white; reverse white at the margin, dark brown at the center.

Material examined: China, Yunnan Province, Lancang, Lahu Autonomous Prefecture, Hani (22°24.381′ N, 100°06.647′ E, elevation 900 m), on dead woody twigs of *S. rarak*, 23 March 2020, G.C. Ren, LGY32 (HKAS 122789, holotype), ex-type culture KUMCC 21-0564; *ibid.*, LGY33 (HKAS 122876, isotype), living culture KUMCC 21-0594.

Notes: *Chromolaenicola sapindi* is introduced as a newly discovered species based on its distinct morphology and analysis of a combined SSU, LSU, ITS, and *tef*1-α dataset. Our samples (KUMCC 21-0564 and KUMCC 21-0594) were clustered with other *Chromolaenicola* species with 84% ML bootstrap and 0.91 BYPP support (Figure 1). Our species can be distinguished from *C. nanensis* and *C. thailandensis* in having 2-celled, guttulate ascospores. Both *C. nanensis* and *C. thailandensis* have muriform ascospores with 3-transverse septa and 1-vertical septum when mature [39]. We did not obtain the asexual morph from *C. sapindi*. Therefore, the morphological comparison between our new species and other *Chromolaenicola* species known only in their asexual morph was not possible. However, based on the phylogenetic distinctiveness, *C. sapindi* is introduced as a new species.

***Dictyoarthrinium*** S. Hughes, Mycological Papers 48: 29 (1952).

*Dictyoarthrinium* was introduced by Hughes [93], with *D. quadratum* as the type species. The genus is characterized by basauxic conidiogenous cell development, conidiophores that are minutely verruculose, subhyaline and transversely septate, conidiophore mother cells which are often hyaline or pale brown and cup-shaped, and conidia of square-to-spherical, subspherical or oblong, pale-to-dark-brown, often 4-celled, and sometimes 16-celled [34,93,94]. Previous studies have accommodated *Dictyoarthrinium* in *Apiosporaceae*, *Sordariomycetes* [91,95,96]. Subsequent studies transferred *Dictyoarthrinium* to *Didymosphaeriaceae*, *Dothideomycetes* based on morphological and molecular evidence [34,70]. Currently, ten species are accepted in *Dictyoarthrinium* [26].

**Figure 5:**
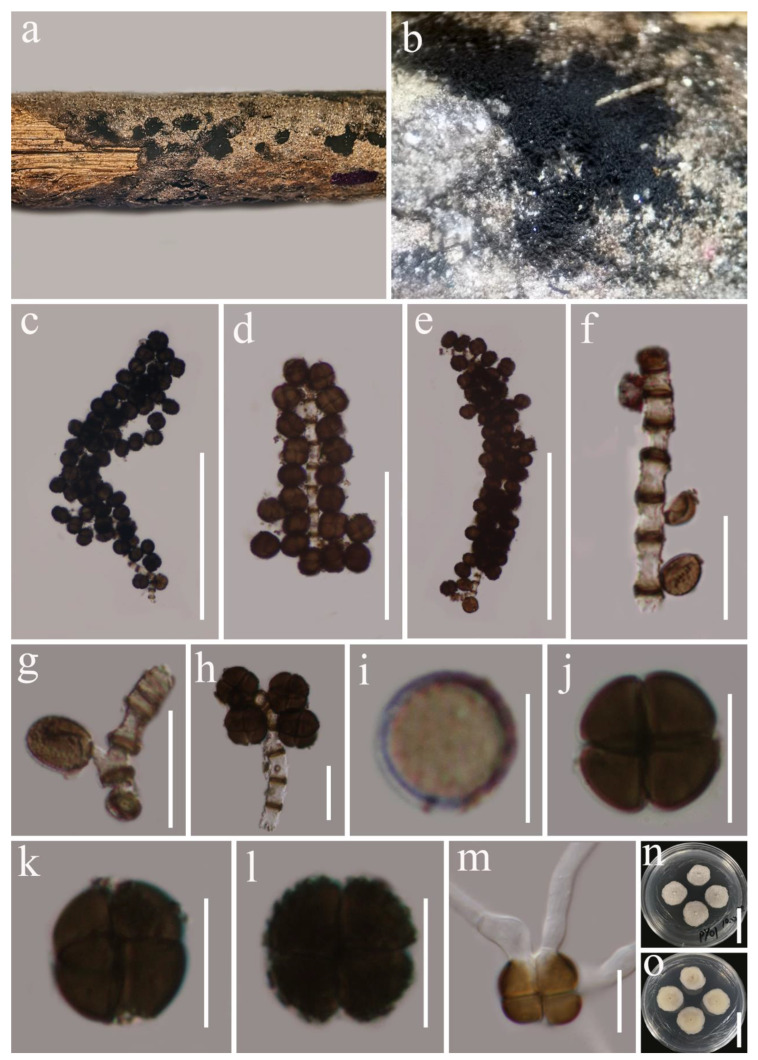
*Dictyoarthrinium thailandicum* (MFLU 22-0040, holotype). (**a**,**b**) Conidia on the host; (**c**–**e**) conidia with conidiophores on stalk; (**f**,**g**) developmental stage of an immature lateral conidium; (**h**) four-celled terminal conidium; (**i**–**l**) warted four-celled mature conidia; (**m**) germinated conidia; (**n**,**o**) culture characters on PDA. Scale bars, (**c**,**e**) 100 μm; (**d**) 50 μm; (**f**–**h**) 15 μm; (**i**–**m**) 10 μm; (**n**,**o**) 30 mm.

***Dictyoarthrinium thailandicum*** G.C. Ren and K.D. Hyde, sp. nov. Figure 5.

Index Fungorum number: IF559807. FacesofFungi number: FoF 10705.

*Etymology*: The epithet “thailandicum” refers to Thailand, where the species was first collected.

Holotype: MFLU 22-0040.

*Saprobic* on dead woody twigs of *Castanopsis* sp. Sexual morph: undetermined. Asexual morph: *Colonies* solitary, irregular, black. *Mycelium* superficial, septate, branched, anastomosing hyphae. *Conidiophores* 130–220 × 4–5 μm ($\bar{x}$ = 180 × 4.5 μm, *n* = 25), erect, macronematous, basauxic, cylindrical, straight or flexuous, sub-hyaline-to-pale-brown, the transverse septa partly brown with distances of 3–7 μm, rough-walled. *Conidiophore mother cells* 4–4.5 × 3.8–4.1 μm ($\bar{x}$ = 4.5 × 4 μm, *n* = 10), cup-shaped, pale brown. *Conidiogenous cells* 3–7 × 3–5 μm ($\bar{x}$ = 5 × 4 μm, *n* = 20), blastic, integrated, terminal and intercalary, cylindrical, sub-hyaline. *Conidia* 9–11 × 8.5–10.5 μm ($\bar{x}$ = 10 × 9.7 μm, *n* = 30), solitary, holoblastic, spherical, 1-celled and sub-hyaline-to-pale-brown when young, cruciate-septate with four cells, constricted at the septa, rounded at the ends, spherical or subspherical, brown-to-dark-brown at maturity, verrucose, mature conidia split along one line of the septa, arising from the lateral or apical part of conidiophores.

Culture characteristics: The colonies on PDA reached 15–20 mm diam. after 14 days at room temperature (25–30 °C), superficial, circular, umbonate at the center, rough surface, with dense mycelia, velvety, flat, and white.

Material examined: Thailand, Chiang Mai Province, Yang Piang Omkoi, on dead woody twigs of *Castanopsis* sp., 25 August 2019, G.C. Ren, YP01 (MFLU 22-0040, holotype), ex-type culture KUMCC 21-0664; *ibid*, Tak Province, Moe Wa Luang Tha Song Yang, on dead woody twigs of *Castanopsis* sp., 17 October 2019, G.C. Ren, TSY02 (MFLU 22-0041, paratype), ex-paratype culture KUMCC 21-0665.

Notes: *Dictyoarthrinium thailandicum* is introduced as a new species based on its distinct morphology and the phylogeny of the combined SSU, LSU, ITS, *tef*1-α dataset. This species is phylogenetically distinct from other *Dictyoarthrinium* species and formed a clade sister to *D. musae* with 76% ML bootstrap and 1.00 BYPP support (Figure 1). This species is similar to *D. musae* in having black colonies, cup-shaped conidiophore mother cells, and cylindrical conidiogenous cells. However, the size of the conidiophores and conidia of *D. thailandicum* (180 × 4.5 µm, 10 × 9.7 μm) is comparatively larger than those of *D. musae* (81.5 × 1.6 μm, 8.7 × 7.9 µm) [34].

***Karstenula*** Speg., Decades Mycologicae Italicae 7–12: no. 94 (in sched.) (1879).

*Karstenula* was introduced with *K. rhodostoma* as the type species [97]. The genus is characterized by globose or sub-globose, black ascomata with flattened apices, and rounded pore-like ostioles. Pseudoparaphyses are cellular and septate; asci are 8-spored, bitunicate, fissitunicate, and cylindrical with short furcate pedicels; ascospores are muriform, ellipsoid-to-fusoid, reddish-brown-to-dark-brown and constricted at the septa [31]. The asexual morph was described by Constantinescu [98] as pycnidial, globose conidioma; enteroblastic, phialidic, determinate, ampulliform-to-doliiform or cylindric-to-ampulliform conidiogenous cells; cylindric, yellow-to-golden-brown conidia with one septate. Twenty-two taxa are listed in species Fungorum [45]; however, molecular data are available only for *K. rhodostoma*. Herein, we introduced another novel *Karstenula* species based on morphology and molecular data.

**Figure 6:**
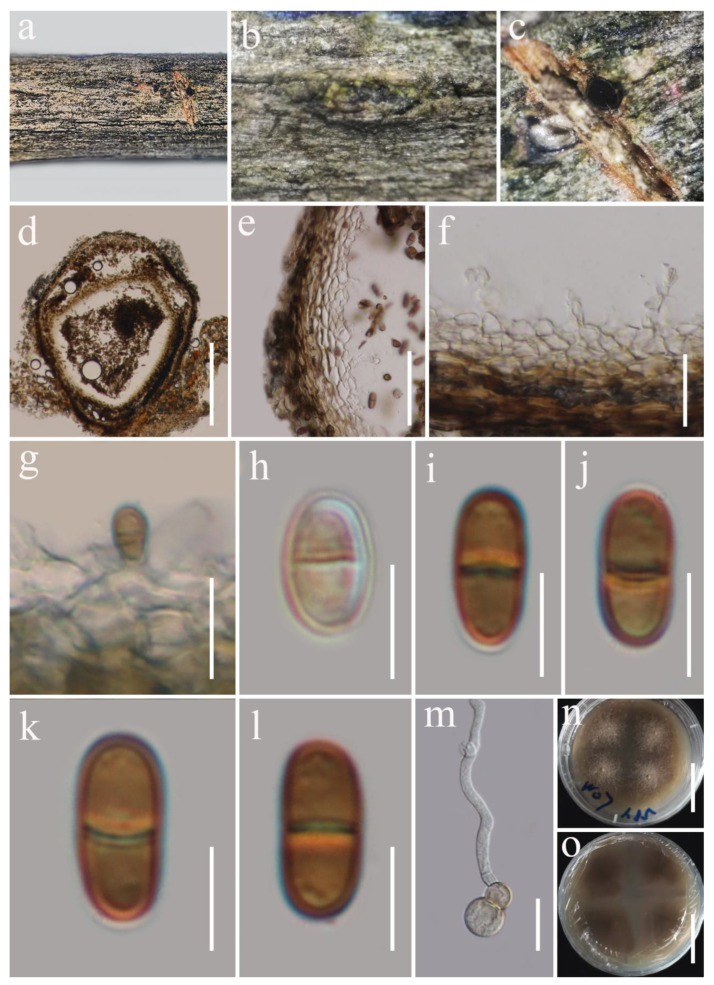
*Karstenula lancangensis* (HKAS 122790, holotype). (**a**–**c**) Conidiomata on the natural wood surface; (**d**,**e**) sections through conidiomata; (**f**) conidioma wall; (**g**) conidiogenous cells and developing conidia; (**h**–**l**) conidia; (**m**) germinated conidium; (**n**,**o**) culture characters on PDA. Scale bars, (**d**) 200 μm; (**e**) 40 μm; (**f**) 20 μm; (**g**) 10 μm; (**h**–**m**) 5 μm; (**n**,**o**) 30 mm.

***Karstenula lancangensis*** G.C. Ren and K.D. Hyde, sp. nov. Figure 6.

Index Fungorum number: IF559808; FacesofFungi number: FoF 10706.

*Etymology*: The species epithet “*lancangensis*” refers to Lancang (Yunnan, China) where the species was collected.

Holotype: HKAS 122790.

*Saprobic* on dead woody twigs of *Cinnamomum glanduliferum*. Sexual morph: undetermined. Asexual morph: *Conidiomata* 270–480 µm high × 240–430 µm diam. ($\bar{x}$ = 410 × 350 µm, *n* = 5), pycnidial, solitary, immersed, unilocular or bilocular, obpyriform, black conidiomata formed under the bark, with broadly rounded apex, and a broad pore opening. *Conidioma wall* 30–40 µm wide, 4–6-layered, composed of an outer layer of brown cells and an inner layer of hyaline cells of *textura angularis*. *Conidiophores* reduced into conidiogenous cells. *Conidiogenous cells* 4–6.5 × 4.4–6.4 μm ($\bar{x}$ = 5.3 × 5.4 µm, *n* = 15), holoblastic, ampulliform-to-doliiform, determinate, hyaline with conspicuous periclinal thickening. *Conidia* 8–10 × 3–4 μm ($\bar{x}$ = 8.8 × 3.6 µm, *n* = 30), oval-to-ellipsoid, straight, aseptate or 1-septate, initially hyaline, becoming brown, cylindrical at maturity, 1-septate (median), partly dark brown septum at median, not constricted at the septum, apex and base rounded, thick-, and smooth-walled.

Culture characteristics: The colonies on PDA reached 45–50 mm diam. after 14 days at room temperature (25–30 °C), superficial, with sparse mycelia, circular, rough, granular, gray-white; reverse dark brown.

Material examined: China, Yunnan Province, Lancang, Lahu Autonomous Prefecture, Hani (22°24.381′ N, 100°06.647′ E, elevation 900 m), on dead woody twigs of *Cinnamomum glanduliferum*, 23 March 2020, G.C. Ren, W07 (HKAS 122790, holotype), ex-type culture KUMCC 21-0670; *ibid.*, W08 (HKAS 122888, isotype), living culture KUMCC 21-0677.

Notes: *Karstenula lancangensis* is introduced as a new species based on its distinct morphology and its phylogenetic position. In the phylogenetic analyses, *K. lancangensis* formed a sister clade to *K. rhodostoma* with 100% ML bootstrap and 1.00 BYPP support (Figure 1). *Karstenula lancangensis* shows similar morphological features to *K. rhodostoma* in having cylindric, 1-septate, brown conidia. However, the size of the conidia of *K. lancangensis* (8–10 × 3–4 μm) is comparatively smaller than those of *K. rhodostoma* (10–) 11–13 (–14) × (4–) 4.5–5 (5.5) µm). In addition, the conidiomata of *K. lancangensis* are unilocular or bilocular, obpyriform with a broadly rounded apex and broad pore, while they are unilocular, globose with a 100 × 80 μm ostiolate in *K. rhodostoma* [98].

***Montagnula*** Berl., Icon. fung. (Abellini) 2: 68 (1896).

*Montagnula* was introduced by Berlese [99], with *M. infernalis* as the type species. Currently, 39 *Montagnula* species are accepted [45], with cosmopolitan distribution [27]. The present paper identified five *Montagnula* isolates from woody plant litter in the GMS. *Montagnula* species are characterized by globose or spherical, immersed ascomata with a clypeus, claviform asci, and fusoid or ellipsoid ascospores with transverse septa and one or more longitudinal septa [31].

***Montagnula donacina*** (Niessl) Wanas., E.B.G. Jones and K.D. Hyde *Index Fungorum* 319: 1 (2017) Figure 7.

≡ *Microthelia donacina* Niessl, Instituto de Coimbra 28: 366 (1881).

≡ *Didymosphaeria donacina* (Niessl) Sacc., Syll. fung. (Abellini) 1: 715 (1882).

≡ *Didymosphaerella donacina* (Niessl) Cooke, Grevillea 18 (no. 86): 29 (1889).

≡ *Munkovalsaria donacina* (Niessl) Aptroot, Nova Hedwigia 60 (3–4): 346 (1995).

Index Fungorum number: IF552762; FacesofFungi number: FoF 04638.

*Saprobic* on decaying wood. Sexual morph: *Ascomata* 320–400 µm high × 350–440 µm diam. ($\bar{x}$ = 350 × 400 µm, *n* = 5), immersed-to-erumpent, solitary or scattered, coriaceous, black, with a central ostiole. *Ostiole* short papillate, 150–190 × 70–90 µm ($\bar{x}$ = 170 × 80 µm, *n* = 5), protruding from substratum. *Peridium* 15–25 µm wide, comprising 4–6 layers of thin-walled, pale-brown-to-brown cells of *textura angularis*. *Hamathecium* comprising 1–2 µm wide, hyaline, cylindrical-to-filiform, septate, branching pseudoparaphyses. *Asci* 70–100 × 10–11 µm ($\bar{x}$ = 87 × 10.7 µm, *n* = 15), bitunicate, fissitunicate, 8-spored, elongate-clavate, slightly curved, with a long pedicel (30–50 µm long; $\bar{x}$ = 40 μm, *n* = 10). *Ascospores* 14–16 × 4.5–6 µm ($\bar{x}$ = 14.5 × 5 µm, *n* = 30), overlapping uni- to bi-seriate, hyaline or yellowish, straight-to-slightly curved, aseptate or 1-septate, guttulate when immature and becoming brown-to-dark-brown when mature, 2-celled, fusiform, rounded ends, 1-septate, constricted at the septum, with slightly pointed upper cell and rounded lower cell, straight to slightly curved, smooth-walled, guttulate, without sheaths or appendages. Asexual morph: undetermined.

Culture characteristics: Colonies on PDA, reaching 90 mm diam. at 14 days at room temperature (25–30 °C), superficial, circular, rough surface, with sparse mycelia, velvety, flat, zonate, white at the margin and center, light brown between margin and center.

Material examined: China, Yunnan Province, Xishuangbanna Dai Autonomous Prefecture, Jinghong, Xishuangbanna Tropical Botanical Garden (21°55.19′ N, 101°15.24′ E), on dead woody twigs of *Ehretia acuminata*, 4 March 2020, G.C. Ren, JH27 (HKAS 122782), living culture KUMCC 21-0579; Thailand, Chiang Rai Province, Mae Yao District, on dead woody twigs of *Betula* sp., 23 September 2019, G.C. Ren, MY22 (MFLU 22-0045), living culture KUMCC 21-0631; Thailand, Tak Province, near Mae Jun river and police school Ban Mea Junta, on dead woody twigs of *Betula* sp., 21 August 2019, G.C. Ren, T404 (MFLU 22-0046), living culture KUMCC 21-0653.

The known hosts are: *Acacia reficiens*, *Acacia* sp., *Adhatoda vasica*, *Ailanthus altissima*, *Annona squamosa*, *Arundo donax*, *Bambusoideae* sp., *Cajanus cajan*, *Calamus australis*, *Careya arborea*, *Citrus aurantiifolia*, *Clerodendrum infortunatum*, *C. multiflorum*, *Coffea arabica*, *C. robusta*, *Dioscorea dumetorum*, *Duranta repens*, *Ficus glomerata*, *Funtumia africana*, *Hibiscus* sp., *Ipomoea carnea*, *Lantana camara*, *Mallotus philippinensis*, *Morus alba*, *Nephelium litchi*, *Nerium odorum*, *Phyllostachys bambusoides*, *Pistacia indica*, *Platanus* sp., *Premna cumingiana*, *Pseudosasa japonica*, *Saccharum officinarum*, *Strophanthus eminii*, *Tectona grandis*, *Terminalia tomentosa*, *Trachycarpus fortunei*, *Wikstroemia* sp., and *Zea mays* [27].

The known distribution is: Australia, Brazil, Central African Republic, China, Colombia, France, Georgia, Hawaii, India, Japan, Louisiana, Myanmar, Namibia, Nigeria, Papua New Guinea, Paraguay, Philippines, Portugal, Sierra Leone, and Tanzania [27].

Notes: Wanasinghe et al. [42] synonymized *Munkovalsaria donacina* and *M. appendiculata* under *Montagnula* based on the phylogenetic analyses of the combined LSU, SSU, and ITS sequence data. Generally, *M. donacina* is characterized by immersed-to-erumpent, single, or gregarious ascomata with a single ostiole, bitunicate, clavate or cylindrical asci with a pedicel and an ocular chamber, ellipsoid, unicellular, 1-septate ascospores strongly constricted at the septum with the upper cell wider and the lower cell rounded [80,100]. The characters of these new isolates (KUMCC 21-0653, KUMCC 21-0579, and KUMCC 21-0631) are similar to *M. donacina* [80]. The multi-gene phylogenetic analysis based on the combined SSU, LSU, ITS, and *tef*1-α sequences showed that our collections (KUMCC 21-0653, KUMCC 21-0579, and KUMCC 21-0631) form a monophyletic group with *M. thailandica* (MFLUCC 17-1508), *M. puerensis* (KUMCC 20-0225, KUMCC 20-0331), *M. donacina* (HFG07004, HVVV01), *M. chromolaenicola* (MFLUCC 17-1469), *M. saikhuensis* (MFLUCC 16-0315), and *M. graminicola* (MFLUCC 13-0352). Based on morphological characteristics and phylogenetic analysis, we report our isolations as the first records of *M. donacina* from decaying wood of *E. acuminata* and *Betula* sp. in Thailand. However, our phylogenetic analyses suggest the presence of a possible complex for *M. donacina*. Hence, extensive studies combining morphology and multi-gene phylogeny of additional samples are necessary.

**Spegazzinia Sacc.**, Michelia 2 (6): 37 (1880).

*Spegazzinia* was introduced by Saccardo [101] with *S. ornata* as the type species. Hyde et al. [95] accommodated *Spegazzinia* in *Sordariomycetes* (*Apiosporaceae*), and based on morphological and molecular evidence, Tanaka et al. [73] transferred *Spegazzinia* to *Didymosphaeriaceae* in *Dothideomycetes*. This was supported by Jayasiri et al. [33], Samarakoon et al. [52], and Thambugala et al. [41]. Currently, 14 taxa are listed in Species Fungorum [45]. *Spegazzinia* is a widely distributed genus with species reported as saprobes on decaying leaves, wood, fruit, and bambusae from Australia, Brazil, China, Cuba, Ghana, and Thailand [38,41,52,102,103,104,105], and endophytes from lichen and leaves in Brazil and India [87,106]. The *Spegazzinia* species have also been reported from the soil in Congo and estuarine sediment [94,107]. Morphologically, most species of *Spegazzinia* have two types of conidia in the same mycelium: α conidia are composed of 4–8 subglobose, very dark cells with very long spines, while β conida are subspherical or broadly ellipsoid in general, flattened in one plane, cruciately septate or muriform, almost always pale brown and smooth [38]. This paper introduces two new isolates of the *Spegazzinia* species observed from decaying wood in terrestrial habitats in China and Thailand.

**Figure 8:**
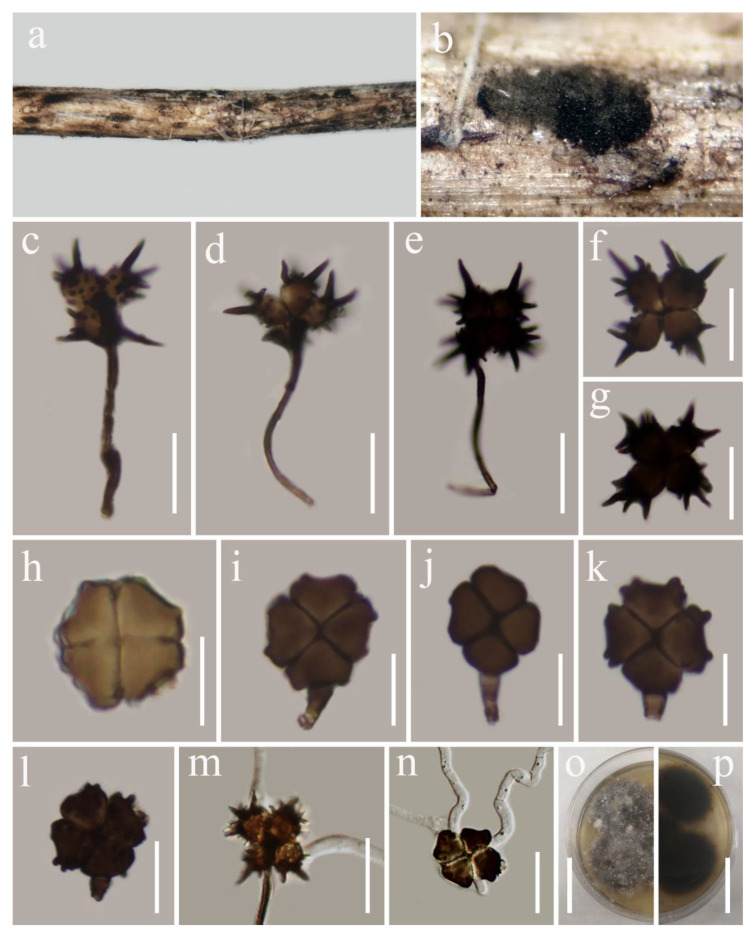
*Spegazzinia jinghaensis* (HKAS 122787, holotype). (**a**,**b**) Fungal colonies on the host surface; (**c**–**e**) conidiophore of *α* conidia and *α* conidia; (**f**,**g**) *α* conidia; (**h**–**l**) β conidia; (**m**,**n**) germinated conidia (**m** *α* conidium, **n** β conidium); (**o**,**p**) culture characters on PDA. Scale bars, (**c**–**e**) 20 μm; (**f**,**g**) 15 μm; (**h**–**l**) 10 μm; (**m**,**n**) 20 μm; (**o**,**p**) 30 mm.

***Spegazzinia jinghaensis*** G.C. Ren and K.D. Hyde, sp. nov. Figure 8.

Index Fungorum number: IF559809; FacesofFungi number: FoF 10707.

*Etymology*: The species epithet “jinghaensis” refers to Jingha (Yunnan, China), the location where the holotype was collected.

Holotype: HKAS 122787.

*Saprobic* on dead woody twigs *Myristica yunnanensis*. Sexual morph: undetermined. Asexual morph: Hyphomycetous. *Sporodochia* dark, dense, dry, powdery, velvety, 2–3 mm in diameter. *Conidiogenous cells* basauxic, ampulate, 5–6 μm high × 4–5 μm wide ($\bar{x}$ = 5.5 × 4.5 μm; *n* = 10), subspherical, hyaline-to-light-brown. *Conidiophores* of α *conidia* up to 80–120 × 1.4–2.0 μm ($\bar{x}$ = 100 × 1.7 μm, *n* = 10), erect or flexuous, unbranched, dark brown. *Conidiophores* of β conidia 3.5–8 × 2.5–3.5 μm ($\bar{x}$ = 5.2 × 3 μm, *n* = 10) short, erect, unbranched, sub-hyaline or light brown. *α conidia* 16–20 × 15–19 μm ($\bar{x}$ = 17.9 × 17.5 μm; *n* = 20), 4-celled, stellate-shaped, brown-to-dark-brown, each cell globose to subglobose with dark brown warts on the surface of the cells, conspicuous spines 3.5–8 × 1–2 μm ($\bar{x}$ = 6 × 1.4 μm; *n* = 15), deeply constricted at the septa. *β conida* 12–16 × 13–17.5 μm ($\bar{x}$ = 14.3 × 15 μm; *n* = 30), 4-celled, disc-shaped, quadrangular or subspherical, initially pale brown, becoming brown-to-dark-brown at maturity, each cell turbinate, crossed septate, the cross-septate partly brown, smooth to verrucose, sometimes cells have raised verrucose around their edges, deeply constricted at the septa, flat from the side view, frequently with attached conidiogenous cells when splitting from the conidiophores.

Culture characteristics: The colonies on PDA reached 30–40 mm diam. at 14 days at room temperature (25–30 °C), superficial, circular, rough surface, gray on the base, with sparse white mycelia on the surface; reverse black.

Material examined: China, Yunnan Province, Xishuangbanna Dai Autonomous Prefecture, Jinghong, Jingha (21°78.06′ N, 101°05.61′ E), on dead woody twigs of *Myristica yunnanensis*, 4 March 2020, G. C. Ren, JHD24 (HKAS 122787, holotype), ex-type culture, KUMCC 21-0495; *ibid.*, JHD25 (HKAS 122878, isotype), living culture, KUMCC 21-0496.

Notes: *Spegazzinia jinghaensis* is introduced as a new species based on its distinct morphology and the combined phylogeny of SSU, LSU, ITS, and *tef*1-α. In the phylogenetic analyses, *S. jinghaensis* is distinct from other sequenced species within this genus and closely related to *S. bromeliacearum* (URM 8084) and *S. intermedia* (CBS 249.89) with strong statistical support (78% ML bootstrap and 1.00 BYPP, Figure 1). *Spegazzinia jinghaensis* differs from *S. bromeliacearum* and *S. intermedia* in having two types of conidia (stellate-shaped conidia: 17.9 × 17.5 μm and disc-shaped conidia: 14.3 × 15 μm). In contrast, *S. bromeliacearum* has globose conidia (26.5–28 μm diam.) with spines, and *S. intermedia* has disc-shaped conidia (18–28 μm diam.), which are dentate at the margin [87,108].

## 4. Discussion

*Didymosphaeriaceae* contains a wide range of taxa occurring on diverse hosts worldwide [26,29,81]. It has been relatively well-studied in recent years, and numerous genera and species are accepted in this family based on phylogenetic studies [30,33,34,42,63,67,77,78,85,109]. Out of 33 genera, *Kalmusia*, *Montagnula*, *Paraphaeosphaeria*, *Paraconiothyrium*, *Phaeodothis*, *Pseudocamarosporium*, *Pseudopithomyces*, and *Spegazzinia* are well studied compared to other genera in *Didymosphaeriaceae*, but many species are likely awaiting discovery [77]. *Barria*, *Cylindroaseptospora*, *Kalmusibambusa*, *Lineostroma*, *Neptunomyces*, *Vicosamyces*, and *Xenocamarosporium* are still monotypic [45], and new species discovery is expected [77]. In this study, we added taxonomic novelties from the GMS to better understand the morphological and phylogenetic relationships of *Didymosphaeriaceae*.

*Septofusispora*, typified by *S. thailandica*, is introduced to accommodate terrestrial dothideomycetes species with a characteristic morphology compared to the extant genera (*Alloconiothyrium*, *Kalmusibambusa*, *Kalmusia*, and *Xenocamarosporium*) in *Didymosphaeriaceae*. This genus is characterized by its clavate asci, fusiform, guttulate ascospores with 4–5 transverse septa, whereas *Kalmusia* has ovoid-to-clavate, 3-septate ascospores (sometimes muriform), with a mucilaginous sheath. *Kalmusibambusa* differs from *Septofusispora* by having ellipsoidal-to-fusiform, 3-septate ascospores with a wide mucilaginous sheath [41,81,90,91]. *Alloconiothyrium* and *Xenocamarosporium* are known only from their asexual morphs [28,63]; therefore, they cannot be morphologically compared with the teleomorph of *Septofusispora*. The phylogenetic analyses showed that *Septofusispora* is distinctly separated from its closely related taxa in this family. Therefore, based on morphological characters and the SSU, LSU, ITS and *tef1-α* sequence data, we recognize *Septofusispora* as a new genus in the family *Didymosphaeriaceae*.

*Alloconiothyrium* was introduced by Verkley and coauthors [28] with *A. aptrootii* as the type species, which is characterized by having pycnidial or eustromatic conidiomata, holoblastic, annellidic conidiogenous cells, olivaceous-brown and irregularly outlined conidia with a rough surface [28]. However, Ariyawansa and coauthors [63] introduced *Alloconiothyrium camelliae* as a new species with uni-loculate, globose-to-subglobose conidiomata, ampulliform-to-doliiform or cylindrical conidiogenous cells and smooth-walled conidia. Furthermore, the multi-gene phylogenies of Ariyawansa and coauthors [63] and our multi-gene phylogenies show that *Alloconiothyrium aptrootii* is well-separated from *A. camelliae.* Therefore, we suggest that *A. camelliae* is a monotypic genus of *Didymosphaeriaceae.* Further studies are needed for a better understanding of the morphological and phylogenetic relationships of *Alloconiothyrium.*

*Karstenula* is an ambiguous genus that exhibits morphological similarities with different families [29,31]. Usually, the sexual morph of *Karstenula* was thought to be characterized by having cylindrical or clavate asci, and brown ascospores with transverse septa and sparse longitudinal septate as dominant characters [31]. For instance, *Karstenula adenocarpi* has oblong ascospores with three transverse septa and 1–several longitudinal septa [110]; *Karstenula calligoni* has clavate asci, and fusoid ascospores with 5–7 transverse septa and a longitudinal septum [111]; *Karstenula guttulata* has cylindrical asci, and ellipsoid, oblong-to-oval ascospores with 4–6 transverse septa and 1–2 longitudinal septa [112]; *Karstenula rhodostoma* has cylindrical asci, and ellipsoid ascospores with three transverse septa and a vertical septum in one or two central cells [31]. As mentioned by Constantinescu [98], the anamorph of *Karstenula rhodostoma* is identical to the coelomycete *Microdiplodia frangulae*, characterized by ampulliform-to-doliiform or cylindric-to-ampulliform conidiogenous cells, and cylindrical, yellow-to-golden-brown conidia with one septum. *Karstenula lancangensis* is similar to the asexual morph of *Karstenula rhodostoma* in having cylindric, 1-septate, brown conidia. However, in our phylogenetic analysis, *Karstenula lancangensis* forms a well-supported clade sister to *K. rhodostoma* in *Didymosphaeriaceae* (Figure 1). Our study provides a reference for further understanding the asexual morphology of *Karstenula.*

*Montagnula donacina* is a prevalent species distributed almost all over the world. It has been isolated from 38 plant species within 24 families [27,80], from which 20 hosts have been from India. However, *M. donacina* has been rarely reported in the GMS, with only two hosts (*Althaea rosea* and *Trachycarpus fortunei*) recorded from China and one host (*Nephelium litchi*) from Myanmar [100,113,114]. The present study reports two *M. donacina* collections from the hosts *E. acuminata* and *Betula* sp. in China and Thailand (the first report of this species in this country). The morphological comparisons between species of *Montagnula,* putatively related to *M. donacina* (Table 2), showed that the ascospores of *M. graminicola* are light brown, verruculose, and have a mucilaginous sheath without guttules. In contrast, all the other species in this clade have no significant apomorphic morphological traits, except for slight differences in the size of ascomata, asci, or ascospores (Table 2), which can be due to ecological factors [115]. In addition, as mentioned in the notes of *M. donacina*, these species are not significantly distinct in phylogeny. Therefore, based on the current morphological data and phylogenetic analyses, we suggest that *M. chromolaenicola*, *M. puerensis*, *M. saikhuensis*, and *M. thailandica* can be considered conspecific with *M. donacina.* However, even though *M. donacina* is widely reported from different hosts, molecular data from only two collections are available in GenBank. Therefore, more extensive studies are needed, applying a combination of different species delimitation criteria to more sequence data obtained from additional samples [116,117] in order to resolve and define the species boundaries in the *Montagnula donacina* complex. Finally, *Montagnula jonesii* was introduced by Tennakoon et al. [49] based on morphology coupled with the analysis of the combined LSU, SSU, ITS, and *tef*1-α sequence data. Despite the fact that our phylogenetic tree based on multigene data showed that *Montagnula jonesii* is not monophyletic with the taxa in *Montagnula* s. str., single-gene analyses (not shown) showed that LSU, SSU, and *tef*1-α data support that *M. jonesii* belongs in *Montagnula*, and only ITS suggests a different placement. Therefore, we believe that the published ITS sequence of *M. jonesii* may be incorrect and needs to be conducted again.

## Figures and Tables

**Figure 3:**
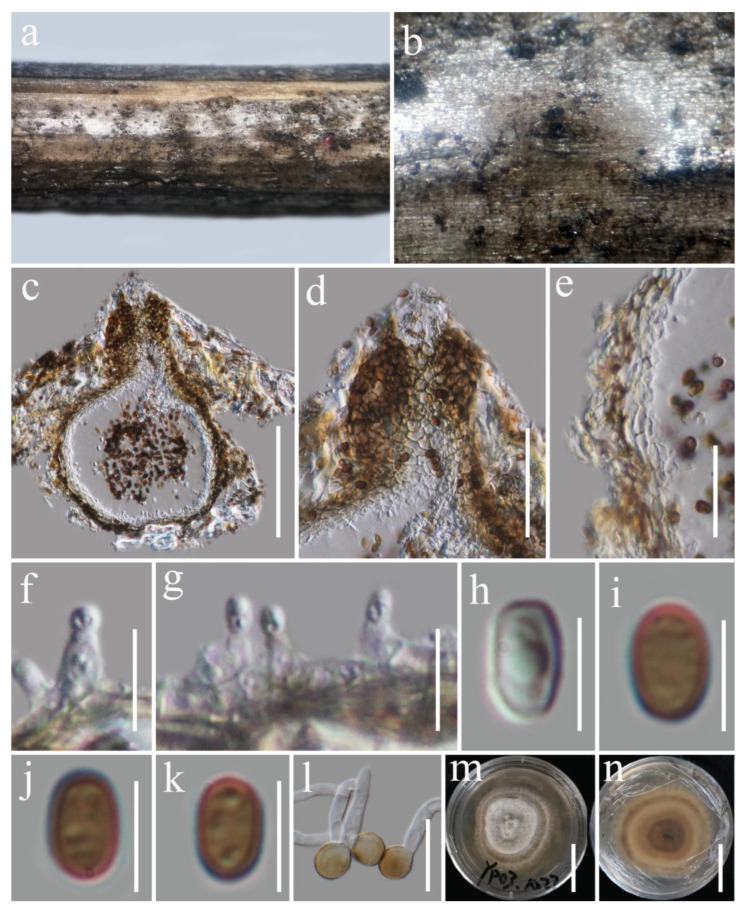
*Austropleospora archidendri* (MFLU 22-0042). (**a**,**b**) Conidiomata on the natural wood surface; (**c**) section through a conidioma; (**d**) ostiolar neck; (**e**) pycnidial wall; (**f**,**g**) conidiogenous cells and developing conidia; (**h**–**k**) conidia; (**l**) germinated conidia; (**m**,**n**) culture characters on PDA (**n** from the bottom). Scale bars, (**c**) 100 μm; (**d**) 50 μm; (**e**) 25 μm; (**f**,**g**,**l**) 10 μm; (**h**–**k**) 5 μm; (**m**,**n**) 30 mm.

**Figure 7:**
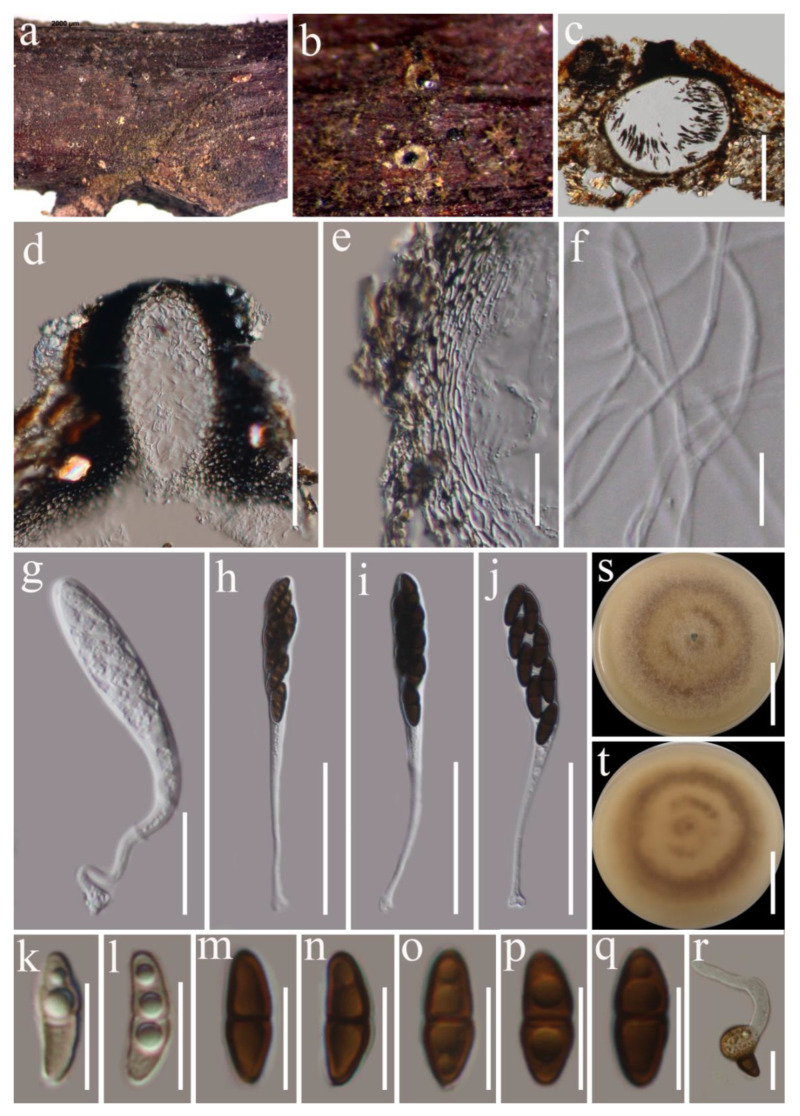
*donacina* (HKAS 122782). (**a**,**b**) Appearance of ascomata on host substrate; (**c**) section of ascoma; (**d**) ostiolar neck; (**e**) peridium; (**f**) hamathecium; (**g**–**j**) asci; (**k**,**l**) immature ascospores; (**m**–**q**) mature ascospores; (**r**) germinated ascospore; (**s**,**t**) culture characters on PDA (**s** from above, **t** from below). Scale bars, (**c**) 200 μm; (**d**,**h**–**j**) 50 μm; (**e**,**g**) 20 μm; (**f**,**k**–**r**) 10 μm; (**s**,**t**) 30 mm.

**Table 2:** Synopsis of the morphological characteristics of *Montagnula* species.

Species Name	Ascomata	Asci	Ascospores						Reference
			Color	Shape	Size	Rows of Ascospores in Asci	Septation	Surface	
*M. chromolaenicola*	Uni-loculate, 310 × 275 μm diam.	90 × 12 μm, bitunicate, elongate-clavate, 8-spored, long pedicellate	Brown to dark brown	Broadly fusiform to ellipsoid	15.5 × 6 μm	Overlapping 1–2-seriate	1	Guttulate	[39]
*M. donacina*	Multi-loculate, 500 μm diam.	90–100 × 12–13 μm, bitunicate, clavate, 8-spored, long pedicellate	Brown	Ellipsoid	14.8–15.2 × 7.5–7.7 μm	Irregularly biseriate	1	Guttulate	[80,100]
*M. donacina* (HKAS 122778)	Uni-loculate, 490 × 410 µm diam.	110 × 13 µm, bitunicate, elongate-clavate, slightly curved, 8-spored, long pedicellate	Pale brown to brown	Broadly fusiform	15 × 5 µm	Overlapping 1–2-seriate	1	Guttulate	This study
*M. donacina* *graminicola*	Uni-loculate, 37–117.22 μm diam.	81.3 × 10.1 μm, bitunicate, cylindrical to clavate, 8-spored, long pedicellate	Brown	Ellipsoid	11.3 × 4.9 μm	Biseriate	1	Verruculose, mucilaginous sheath	[81]
*M. puerensis*	Uni-loculate, 300–600 × 230–380 μm diam.	92 × 11 μm, bitunicate, elongate-clavate, 8-spored, long, furcate pedicellate	Brown to dark brown	Ellipsoid	14 × 6 μm	Biseriate	1	Guttulate	[83]
*M. saikhuensis*	Uni-loculate, 411.7 × 460.5 μm diam.	84.2 × 11.2 μm, bitunicate, elongate-clavate to short cylindrical, 8-spored, long pedicellate	Brown to blackish	Ellipsoid	14.6 × 5.1 μm	Overlapping 1–2-seriate	1	Guttulate	[42]
*M. thailandica*	Uni-loculate, 380 × 340 μm diam.	90 × 11 μm, bitunicate, elongate-clavate, slightly curved, 8-spored, long pedicellate	Brown to reddish-brown	Broadly fusiform to ellipsoid	15 × 5.5 μm	Overlapping 1–2-seriate	1	Guttulate	[39]

## Data Availability

The datasets generated for this study can be found in the GenBank, NCBI and the accession numbers are given in Table 1. Newly introduced fungal names were registered at the Index Fungorum and the identification numbers are shown in their respective entries.
